# Advances in the Detection of Dithiocarbamate Fungicides: Opportunities for Biosensors

**DOI:** 10.3390/bios11010012

**Published:** 2020-12-30

**Authors:** Pablo Fanjul-Bolado, Ronen Fogel, Janice Limson, Cristina Purcarea, Alina Vasilescu

**Affiliations:** 1 Metrohm DropSens, S.L., Vivero de Ciencias de la Salud, 33010 Oviedo, Spain; pablo.fanjul@metrohm.com; 2 Biotechnology Innovation Centre, Rhodes University, Makhanda 6140, South Africa; r.fogel@ru.ac.za (R.F.); j.limson@ru.ac.za (J.L.); 3 Institute of Biology, 296 Splaiul Independentei, 060031 Bucharest, Romania; cristina.purcarea@ibiol.ro; 4 International Centre of Biodynamics, 1B Intrarea Portocalelor, 060101 Bucharest, Romania

**Keywords:** dithiocarbamate fungicides, chromatography, Raman spectroscopy, sensors, enzyme inhibition, voltammetry, biosensors

## Abstract

Dithiocarbamate fungicides (DTFs) are widely used to control various fungal diseases in crops and ornamental plants. Maximum residual limits in the order of ppb-ppm are currently imposed by legislation to prevent toxicity problems associated with excessive use of DTFs. The specific analytical determination of DTFs is complicated by their low solubility in water and organic solvents. This review summarizes the current analytical procedures used for the analysis of DTF, including chromatography, spectroscopy, and sensor-based methods and discusses the challenges related to selectivity, sensitivity, and sample preparation. Biosensors based on enzymatic inhibition demonstrated potential as analytical tools for DTFs and warrant further research, considering novel enzymes from extremophilic sources. Meanwhile, Raman spectroscopy and various sensors appear very promising, provided the selectivity issues are solved.

## 1. Introduction

**Figure 1:**
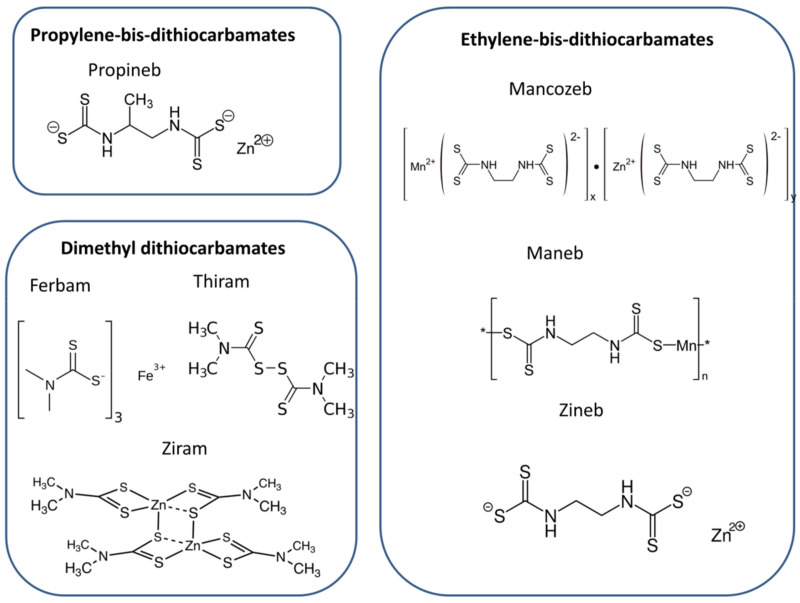
Classification of dithiocarbamate fungicides (DTF) and chemical structures of main representatives from each group.

Dithiocarbamate fungicides (DTFs) are non-systemic pesticides that have been used since the 1940s to control a number of fungal diseases in various crops and ornamental plants. Propineb, zineb, maneb, thiram, and mancozeb are amongst some of the most well-known and used fungicides, the chemical structures of which are presented in Figure 1.

**Figure 2:**
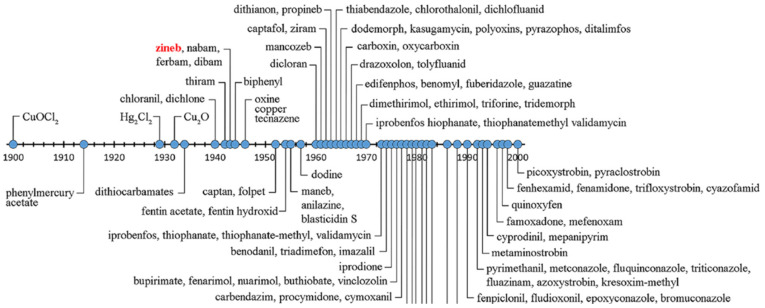
Timeline of the development of fungicides, showing the appearance of DTFs from 1930s onwards. Reproduced from [1], with permission.

While the development of new pesticide molecules and formulations has continued over the years, currently used DTFs such as maneb, mancozeb, propineb, thiram, and ziram were introduced more than 50 years ago (Figure 2) [1]. Mancozeb, propineb, and thiram are among the top selling fungicides, e.g., mancozeb sales are expected to reach $18 billion by 2025 [2].

Based on their chemical structure, DTFs are classified as propylene-bis-dithiocarbamates (PBs, e.g., propineb), ethylene-bis-dithiocarbamates (EBs, e.g., mancozeb, maneb, and zineb), and dimethyl dithiocarbamates (DDs, e.g., thiram, ziram, and ferbam). The DTFs from different groups have different toxicity, resulting in variances in the risk assessment for exposure to specific fungicides. Due to their toxicity, fungicides like zineb were banned in many countries around the globe, including the US and the EU, while in countries where it is currently allowed for use, maximum residue limits in the range of ppm are imposed by various organizations worldwide, for various food and agricultural products. The European Commission established maximum residue limits (MRL) of 0.01–25 ppm for dithiocarbamates in various plants and products of vegetable or animal origin (expressed as CS_2_, including maneb, mancozeb, metiram, propineb, thiram, and ziram) [3]. Excessive use of DTFs has continued in recent years, e.g., amounts higher than the MRL have been detected in tomatoes [4], kiwi, and pears [5], etc.

Dithiocarbamate pesticides have low solubility in water and a number of organic solvents; hence, they are not typically extracted and analyzed by the multiresidue chromatographic methods used for screening other pesticides. This limitation, coupled to the wide number of DTF compounds applied as pesticides (Figure 2) meant that the simplest method for the analysis of dithiocarbamates relies on their degradation in acid media and analysis of the resulting CS_2_ by spectrophotometric or chromatographic methods [3]. A major drawback to the detection of DTFs by quantification of formed CS_2_ is the lack of specificity, as this does not allow the identification of parent DTFs present within the sample. Additionally, the analysis of DTFs based on CS_2_ is affected by false positive results in agricultural products containing high levels of organic sulfur compounds: notably, CS_2_ produced in acidic media by *Brassicaceae* and *Alliaceae* vegetables (e.g., cabbage and onion) was identified [3].

In a 2017 report, the European Food Safety Agency EFSA reiterated the need to develop specific analytical procedures for each active substance in this group of fungicides [5]. Currently, specific, single residue methods are available for thiram, propineb, and ziram [3].

Details of the various analytical procedures available for the determination of DTFs are given below, together with a discussion of the recent progress and perspectives in this field. A particular focus is placed on sensors and biosensors with the potential of delivering simple and fast detection.

## 2. Advances in DTFs Detection

### 2.1. Standard Chromatographic Methods for DTFs Detection

As with many other pesticide classes, the most selective methods for the determination of DTFs are based on gas chromatography coupled with-mass spectrometry (GC-MS) [6] and on reverse phase liquid chromatography coupled with optical, electrochemical, or mass-spectrometry detectors [7,8,9,10].

Nakamura et al. described a GC-MS method for the detection of 10 dithiocarbamates in foods [6]. The compounds were extracted as water-soluble sodium salts with cysteine and ethylenediaminetetraacetic acid (EDTA) and then were derivatized by methylation. This strategy enables differentiation between the DTFs from different groups, i.e., thiram, ziram, ferbam, zineb, maneb, mancozeb, milneb, metiram, propineb, nickel bis(dithiocarbamate), and polycarbamate. The quantification limits, expressed as CS_2_ were 0.01 mg/kg in brown rice, soybean, potato, spinach, cabbage, apple, orange, pumpkin, cacao, cattle muscle, cattle fat, cattle liver, salmon, eel, milk, chicken egg, honey, and shrimp and 0.1 mg/kg in green tea samples [6].

An alternative approach based on reverse-phase liquid chromatography circumvented the need for derivatization [10], being based on the formation of ion pairs between the DTFs anions and tetrabutylammonium cations in alkaline media containing EDTA. The approach used two detectors connected in series, UV and electrochemical, achieving limits of quantitation, of 9, 12, 8 and 12 µg/L CS_2_ for N-methyl-DTF, N,N-dimethyl-DTF, ethylenebis-DTF, and propylenebis-DTF [10]. However, while this method appears simpler and provided good recoveries from spiked fruit and tomatoes, accurate results can only be obtained for surface-intact vegetables.

Chromatographic methods developed with the aim of achieving sensitive and selective detection of DTFs relied not only on mass-spectrometry, UV, and electrochemical detectors but were also coupled with atomic absorption spectrometers. Indeed, the presence of different metals like zinc, manganese, and nickel in the structure of some DTFs makes it possible to identify the DTFs by atomic absorption spectrometry. Thus, procedures requiring high instrumental infrastructure like HPLC-UV with detection at 272 nm coupled with atomic absorption spectrometry were used for the determination of 10 pesticides, and were able to distinguish between zineb, maneb, and mancozeb in diverse matrices [11].

More recently, an LC-MS method was described for the analysis of 10 DTFs in beer, fruit juice, and malt samples, based on the common strategy of transforming the fungicides in water soluble salts and derivatizing them with methyl iodide [8]. The extraction of methylated derivatives of the DTFs was performed using a “quick, easy, cheap, effective, rugged, and safe” (QuEChERS) method, subsequently purifying the extracts by dispersive solid-phase extraction prior to LC-MS analysis. Separation of the methyl derivative DTFs compounds by Ultra Performance Liquid Chromatography (UPLC) used a C_18_ column and the detection was performed by ESI-MS in selected reaction monitoring (SRM), positive ion mode. The quantitation limits reached by this method for three representatives of the main groups of DTFs, i.e., propineb (a propylene-bis-dithiocarbamate), mancozeb (an ethylene-bis-dithiocarbamate), and thiram (dimethyl dithiocarbamate) were 0.52, 0.55 and 6.97 µg/kg, respectively. The procedure was successfully applied for the determination of DTFs in beer and fruit juice. A similar approach but with derivatization to dimethyl derivatives was described by Li et al., who reported detection limits of 0.6–1.6μg/kg and 0.8–2.5 μg/kg for mancozeb and propineb, respectively, in different vegetable food matrices [9].

While the selective detection of relevant DTFs at sensitivities below those required for the ppm MRL established for foodstuffs has been reported for several applicable matrices [7,8,9,10,12,13,14], the main limitations to the use of chromatographic techniques in routine detection of DTFs remain the cost of equipment and the relative complexity of analysis. Despite advances made in the field of portable GC-MS and HPLC chromatographic devices [15], the base costs of this equipment remain important (~US $100,000 or more). Similarly, the reliance of these techniques on purified organic solvents and the complex protocols required to satisfactorily detect DTFs with these approaches, including derivatization, purification, and pre-concentration of samples prior to the analysis (e.g., [8]) may prove prohibitive when attempting to apply these techniques at the scale required for the routine detection of DTFs in agricultural products. In summary, despite general progress in the development of cleaning cartridges, QuEChERS extraction methods and analytical instrumentation for chromatography and MS, there was not a huge advancement in the last years regarding the determination of DTFs.

### 2.2. Spectroscopy-Based Analysis Methods

Raman spectroscopy provides information about the vibrational states of molecules and therefore their functional groups and chemical structure. It is considered a fingerprint technique since each Raman spectrum corresponds to a unique chemical compound and a spectra library repository can provide rapid identification of molecules for analytical purposes. This highly selective technique is balanced by a lack of sensitivity due to the standard weak Raman scattering signals that usually provides detection limits at concentrations in the order of 10^−2^ M.

A great enhancement of the Raman signal can be obtained by the interaction of the analyte with some metallic surfaces mainly made of gold, copper or silver. Surface enhanced Raman scattering (SERS) was firstly introduced by Fleishman et al. in 1974 working with pyridine as a model probe and an electrochemically roughened silver electrode as surface [16]. The SERS effect increases the interest of Raman spectroscopy in analytical chemistry due to the sensitivity increase that can led towards to the detection of a single molecule [17].

First peak spectra assignments of dithiocarbamates (DMDTC and DEDTC) over silver surfaces (colloids and surfaces) were obtained in the 1990s by Mylrajan [18] and Tse Yuen [19], including FT-IR data.

**Figure 3:**
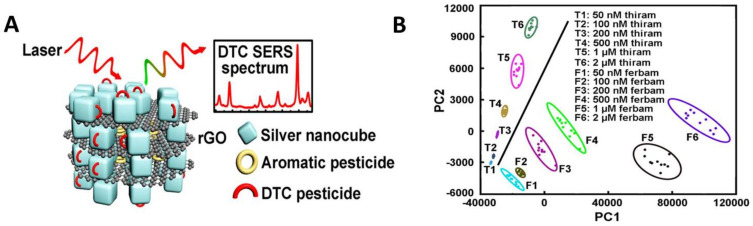
(**A**) Principle of the surface enhanced Raman scattering (SERS) detection of DTF using rGO-wrapped Ag nanocubes. (**B**) Principal component analysis (PCA) plot (PC1 versus PC2) of signals extracted from 10 SERS spectra of thiram and ferbam with concentration ranging from 50 nM to 2 μM. Reproduced from [21] with permission.

For quantitative analysis several works have been done using as analytical signal the strongest band at around 1380 cm^−1^ assigned to the C-N stretching mode and symmetric CH_3_ deformation mode of dithiocarbamates pesticides. Most of these approaches rely on silver or gold based nanomaterials to get the SERS enhancement:

Thiram was analyzed using silver nanoparticles clusters for SERS analysis, reaching a detection limit of 24 ppb (10^−7^ M) that is much lower than the maximum residue limit ranging from 2 to 15 ppm in fruit prescribed by the U.S. Environmental Protection Agency (EPA) [20]. A combination of silver nanocubes with reduced graphene oxide in a sponge-like structure was used for the detection of thiram and ferbam achieving detection limits of 10 and 16 ppb, respectively, based on the intensity of the characteristic signal at 1382 cm^−1^ [21]. In this case (Figure 3A) graphene oxide was used to remove the interference from aromatic pesticides adsorbed to it and allowing the SERS effect in the silver nanocubes to be detected only with DTF pesticides. As SERS spectra of ferbam and thiram were similar in terms of peak location and intensity ratios, principal component analysis (PCA) was used to distinguish which fungicide is present in the environment (see Figure 3B). It should be noted that the tolerances for ferbam residues in pear, apple, grape, mango, cabbage, and lettuce, range from 4 to 7 ppm, much higher concentrations than the ppb level achieved with this approach.

The results revealed that while there is a clear differentiation between the two DTFs based on PCA when analyzing individual spectra, it was not possible to distinguish between them when present in mixtures. Still, the total amount of the S−C−S group in the fungicide mixture was correlated with I^0.5^, where I is the intensity of the SERS signal at 1382 cm^−1^. Other recently published papers related to the detection DTFs usually work with thiram as probe analyte using a variety of silver nanomaterials for the SERS effect [22,23,24].

Gold nanorods were used for ultra-sensitive detection of DTFs [25,26]. Thiram, ferbam, and ziram were determined in the range of low ppb concentrations (10^8^ M). The interaction of the fungicide with the gold substrate is supposed to undergo spontaneous cleavage of their metal–sulfur bonds to produce the dimethyldithiocarbamate ion which then assembles on the substrate surface. Since the degradation processes produce identical ions for all these molecules, the SERS spectra of the three pesticides appear very similar; therefore, multivariate data analysis techniques can be coupled when working with real samples [25]. For additional information of the spontaneous assembly of dithiocarbamate ligands on gold metal substrates, a research paper by Alexander Wei et al. [27] can be revisited. Gold nanoparticles trapped into cellulose matrices has been reported as an interesting solution for the in situ extraction and detection of thiram in residues in soil and fruits [28]. In addition, screen printed gold electrodes roughened through an electrochemical pretreatment arise as an easy to use and portable solution for the detection of thiram and other pesticides as well [29].

Efficient sampling, enabling fast and quantitative recovery of DTFs is a limiting factor for the practical applications of SERS. In this respect significant progress was reported in the last years with regards to flexible substrates that can be brought in close contact with sample surface, with double role of collecting the DTFs from samples and as SERS substrate. This includes an approach for the in situ extraction and SERS substrate formation [28], applied for samples with irregular surfaces such as soil, strawberries, and cucumbers as well as swab-type devices, e.g., applied for sampling thiram from the surface of intact fruits and vegetables [24].

The detection of thiram was demonstrated in a variety of spiked samples, including soil, strawberries, tomato, cucumber, water, etc., for which satisfactory recoveries were calculated.

Raman spectroscopy is one of the best suited techniques for the determination of dithiocarbamates due to the high selectivity provided by the fingerprint spectrum of each molecule and the high sensitivity achieved through the SERS effect. Additionally the potential use of portable analyzers and cost effective disposable SERS substrates allows its application in field analysis. However, some different sensing alternatives based on other optical detection modes are reported showing advantages and drawbacks for dithiocarbamate determination.

Fourier transform infrared (FTIR) spectrometric procedures were developed for the determination of ziram and thiram in solid samples [30] and in vapor phase samples, after ziram decomposition [31]. Both FTIR-based methodologies allowed for pesticide detection in the order of tens and hundreds of mg as absolute detection limits.

From our perspective the rapid evolution of Raman spectrometers from benchtop instruments to portable devices and the cost reduction of the technology makes it very powerful for “in situ” analysis. Moreover the huge number of cost-effective SERS substrates developed using different nanomaterials position this technique as the leader in terms of sensitivity. The intrinsic selectivity provided by the Raman spectrum avoids the need of any other recognition element. However, when several DTFs are present in the same sample, chemometrics methods have to be applied to SERS data in order to specifically detect individual fungicides. Sample collection and sample immobilization in the SERS substrate to get quantitative analysis are still the main drawbacks to be solved.

### 2.3. Optical and Electrochemical Assays

Electrochemical sensors and optical assays have been investigated as simpler, miniaturized, and cost-effective analytical devices that could represent viable alternatives to the chromatographic methods and Raman spectroscopy.

#### 2.3.1. Electrochemical Sensors

**Figure 4:**
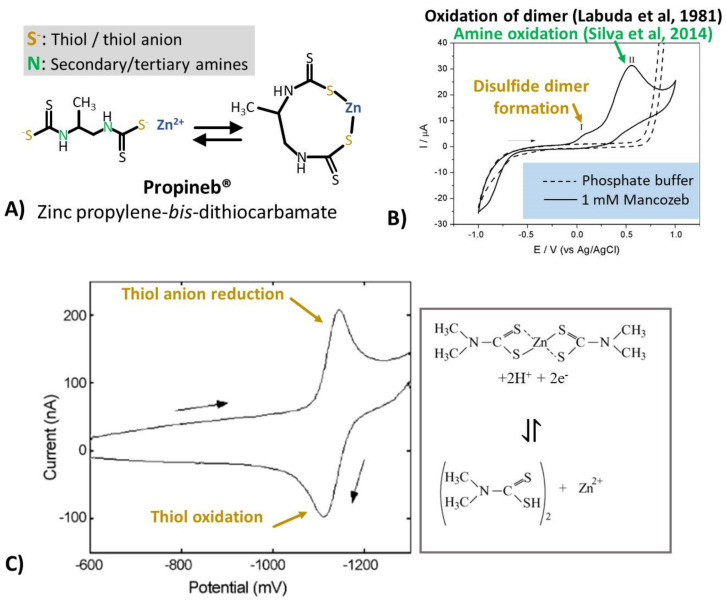
Electrochemical detection of dithiocarbamate pesticides is facilitated by the multiple electrochemically-active moieties they possess. (**A**) Commonly-reported electroactive moieties of dithiocarbamates, using Propineb^®^ (extracted from [36]) as a general example of this class of compound. (**B**) Cyclic voltammograms of Mancozeb at a Boron-doped diamond electrode in phosphate buffer (extracted from [36]), showing anodic processes involving its thiol and/or amine moieties: two separate possible mechanisms behind the more positive peak are shown, one cited in [36] and that discussed by [35]. (**C**) Cyclic voltammogram of Ziram at a Hanging Mercury Drop Electrode in Britton–Robinson buffer, pH 2.8 (extracted from [37]. Peaks are attributed to the reversible reduction of the thiol moieties and subsequent displacement of the zinc moiety (inset, from same reference) at mercury electrodes. Reproduced from [36,37], by permission).

The electrochemical detection of dithiocarbamate fungicides has been well-known for several decades [32,33], facilitated by the multiple electroactive sites present on these compounds (Figure 4A).

Under aqueous conditions, detection via the thiol groups is facilitated by the dissociation of metal-complexed dithiocarbamates to produce carbamate anions (Figure 4A) [34]. At mercury electrodes, carbamate anions are readily reduced to form mercury complexes (e.g., Figure 4B). At inert electrodes (such as carbon and platinum), carbamate anions are broadly irreversibly oxidized (e.g., Figure 4C) by a monoelectron step [33]. This forms radical intermediates that frequently dimerize via the sulfur atoms to form disulfide products [33]; this complex being further oxidized at higher anodic potential [33,35].

Apart from the particular structure of dithiocarbamate under investigation, the exact detected moiety/ies can be controlled by the selection of experimental parameters, such as electrode type, electrolyte solvent selection (especially pH) and the electrochemical waveform applied to their detection. This has facilitated the detection of numerous commercially-used dithiocarbamates in multiple studies (Table 1), in both laboratory-formulated pure standards and within real samples (predominantly commercial formulations of the pesticides, and pesticide-spiked foodstuffs).

As is evident by the large number of recent publications, the application of electrochemical methods of detection of dithiocarbamates remains an actively-researched field of enquiry. This is due to a combination of their aforementioned ready detection via electrochemical means and the inherent sensitivity of electrochemical detection methods. A number of publications report the sensitive detection of different dithiocarbamate fungicides: analytical quantification ranges of reports are frequently in the µM-to-nM orders of magnitude and reported limits of detection often in the nM range (Table 1).

The large variation in the dithiocarbamates studies, the working electrodes and waveforms in the literature reviewed makes direct comparison across all the entries difficult, but nonetheless, some trends are evident across the literature.

At high concentrations of unionized forms of DTC pesticides, the low aqueous solubility remained a continual analytical challenge. Most concentrated stocks were formulated in the μg/mL range, typically achieving this by including chelating agent, most often EDTA, e.g., [36,51] and high pH in aqueous solvents to improve solubility; sometimes with combinations of the two [59]. Others make use of organic solvents: acetonitrile (e.g., [56], chloroform [59], methanol [12,39,40,42], and ethanol [47] to solubilize these.)

The low aqueous solubility of these pesticides somewhat improves sensitivity by electrochemical methods, due to the tendency of target molecules to adsorb to the surface of electrodes, effectively preconcentrating the analyte before quantification. To capitalize on this phenomenon, most reported investigations explicitly make use of adsorptive waveforms (Table 1): dithiocarbamates have been routinely detected using adsorptive stripping voltammetry waveforms, e.g., [51], cathodic stripping voltammetry [43], anodic stripping voltammetry [45] or other forms that deliberately include an adsorptive step prior to analysis (e.g., pulsed amperometric detection by [36]. The majority of electrode surfaces selected tend to be those extensively used for adsorptive studies: aromatic and graphitized carbon preconcentrating the pesticides using the hydrophobic nature of the ethylene backbones. A more specific targeting of the thiol moieties of thiocarbamates has been reported, either using gold electrodes (e.g., [54] to form thiol-gold self-assembled adlayers at the electrode surface or the formation of mercury amalgams at mercury drop-electrodes [34].

As previously discussed, mercury drop electrodes were frequently employed for the detection of dithiocarbamates. All of the identified studies made use of cathodic stripping voltammetry to detect and quantify their investigated dithiocarbamates, preceded by an adsorptive preconcentration. Several of these reports explicitly cited the reduction of thiol moieties as the basis for signal generation at these electrodes [37,44,49]. A similar catalytic effect using copper–mercury amalgam electrodes was reported for monitoring of thiram [49]. Despite the overall sensitivity of this approach (the majority of studies reporting limits of detection in the low nM range), the use of mercury electrodes in routine electrochemistry has declined in recent years, due to the associated health and environmental hazards of this metal and may preclude its use in the commercial detection of these fungicides.

The detection of dithiocarbamates by unmodified carbon electrode surfaces (i.e., entries reported to use boron-doped diamond, glassy carbon electrodes, carbon-paste electrodes) form the largest group found during this review. Similar to expectations, these predominantly use anodic processes to detect their targets, for reasons discussed previously. While inherently less sensitive than the cathodic approach for mercury drop electrodes (stated limits of detection using voltametric approaches with carbon surfaces typically reported at µM levels in Table 1), optimization of the detection waveform used and other experimental conditions can enhance sensitivity to the nM level (e.g., [48,59] in Table 1), similar to those achieved using mercury drop electrodes.

Many of the detection methods investigated using unmodified metal and carbon electrodes share a disadvantage: the application of substantial cathodic or anodic voltages required to effectively detect dithiocarbamates. These substantially decrease the signal specificity used, as numerous other compounds capable of electrochemical reactions at more negative working electrode potentials (for anodic detection) or more positive (for cathodic detection) will also contribute signal and decrease selectivity. Several of these reports [59] reported that operating at electrode potentials where Faradaic processes in the electrolyte occurred began affecting signal-to-noise ratios due to this limitation. Despite this, numerous reports in Table 1 also include samples extracted from foodstuffs, soil and river water samples and report satisfactory detected recoveries from these using the above analytical approaches.

To further improve selectivity, reverse phase liquid chromatographic separation of sample components has been reported as a means of coupling the sensitivity of amperometric detection using unmodified electrodes with some means of separating out the various components of the samples (Table 2). This allowed for several investigations to separately measure dithiocarbamates in a mixture of these pesticides.

Increasingly, possibly as a sustainable alternative to mercury electrodes, the more recent trend is the investigation of catalysts and/or electrode modifiers to improve signal specificity or sensitivity [50] of the assays. Some of these forms of modification attempt to enhance the adsorption of dithiocarbamates to enhance detection sensitivity e.g., PTFE [41,42] and mercury amalgams [49]. Others apply catalysts to facilitate electron-transfer between DTFs and the electrode: the most commonly-identified are metallophthalocyanines [45,46] and metal nanoparticles [55,57] to catalyze anodic detection of DTFs. While all of the catalysts reportedly impart enhanced sensitivity, none of them increase specificity: the broad electrocatalytic effects of metallophthalocyanines [61] and metal nanoparticles [62] are widely-known.

While overall good sensitivity is reported using electrochemical detection of DTFs, neither the electrode surfaces nor the waveforms selected are specific enough for routine detection and are inherently prone to multiple interferences. Despite the recent research focus on alternative catalysts and modifiers, a specific electrochemical DTF chemosensor is not yet apparent in the literature.

#### 2.3.2. Optical Assays

Some simple colorimetric assays working with metallic nanoparticles based on copper [63], silver [64], or gold [65] can be used for the detection of pesticides due to the solution colour change after nanoparticles aggregation in presence of the analyte. In these approaches nanoparticle protective agents such as sodium dodecyl sulfate (SDS), cetyl tributylammonium bromide (CTAB) or citrate play a key role in the procedure that finally allows a semiquantitative detection by naked eye or a quantitative detection by using a simple spectrophotometer. Detection limits in the ppb level can be achieved in samples of environmental interest [65]. Ease of use and no need for expensive analytical instrumentation are the main advantages of this approach; however, a lack of selectivity can be highlighted as the main drawback. A multicolor sensor for visual screening of total dithiocarbamates based on the inhibition of gold nanoparticles growth in presence of the fungicides [66] clearly demonstrates these conclusions. Sufficient sensitivity, and short analysis time makes it useful for screening purposes but the lack of selectivity between ziram, thiram, and zineb makes it only useful for total DTCs detection.

Ziram was quantified with a detection limit of 2nM in tomatoes and rice using the same gold nanoparticle aggregation principle. In this case, the detection was made using a fluorimeter since a yellow fluorescence decrease of quantum dots was monitored because of a quenching mechanism [67]. Working with phosphorescent Mn doped ZnS quantum dots a quenching effect was also used for the detection of thiram at a detection limit of 50 nM. This chemosensor was tested in fruit peels and minor interferences were found with atrazine [68].

There are some other works related to the detection of thiram by electrochemiluminescence (ECL), but these cannot be considered in the range of sensor devices. Most of them are online analytical methodologies based on Flow Injection Analysis systems where the ECL analytical signal is enhanced in the presence of thiram [69,70]. Working with the same highly sensitive analytical technique (ECL), an ELISA assay was developed for the detection of thiram in honeybees. An indirect competitive assay was implemented and a detection limit of 9 ppb was achieved [71]. According to the state of art, there is still enough scope for the development of optical sensors working in the detection of dithiocarbamates fungicides, mainly combining specific recognition elements for these analytes with an optical transducer. In this case enzymatic, antibody, or aptamer based optical biosensors should be further developed.

### 2.4. Biosensors Based on Enzyme Inhibition

Coupling a specific biorecognition element with a sensitive detection method as is achieved in biosensors goes a long way towards improving the selectivity of detection and eliminating the need for chromatographic separation or complicated chemometric analysis. In addition, compared to electrochemical sensors for DTFs discussed in Section 2.3.1, operating at large overpotentials where interferences in real samples are important, electrochemical biosensors rely on electron transfer mediators or on direct electron transfer from the enzymes to the electrode. Electrochemical biosensors are thus operated at lower overpotentials, alleviating the problems related to nonspecific Faradaic reactions, common for electrochemical sensors.

However, the detection of DTFs was very little explored in the biosensing field compared to other classes of pesticides. While the first ELISA tests for the detection of thiram in a food matrix, i.e., in spiked lettuce was reported 20 years ago [72], no reports of biosensors based on antibodies followed. Moreover, there are no recent reports on specific antibodies and related kits for DTFs, no aptamers have been selected for specific DTFs and no sensors or sample extraction methods based on molecularly imprinted polymers have been reported for this type of pesticides. The only attempts to specifically detect DTFs with biosensors exploited enzyme inhibition and the toxic effects on mammalian cells as detailed in Section 2.4.1 below.

The detection of pesticides based on the principle of enzyme inhibition has long been explored as an alternative to chromatographic and spectroscopic methods. Various enzymes, including acetylcholinesterase, butyrylcholinesterase, alkaline phosphatase, organophosphorus hydrolase, the enzymatic complex of Photosytem II in plants, algae, and cyanobacteria, as well as urease, laccase, tyrosinase, and aldehyde dehydrogenase have been used for the detection of various classes of pesticides [73,74,75,76]. DTFs are known inhibitors of aldehyde dehydrogenase, tyrosinase, and laccase, which led to the development of several biosensors that make use of these enzymes [77,78,79,80,81,82,83].

**Figure 5:**
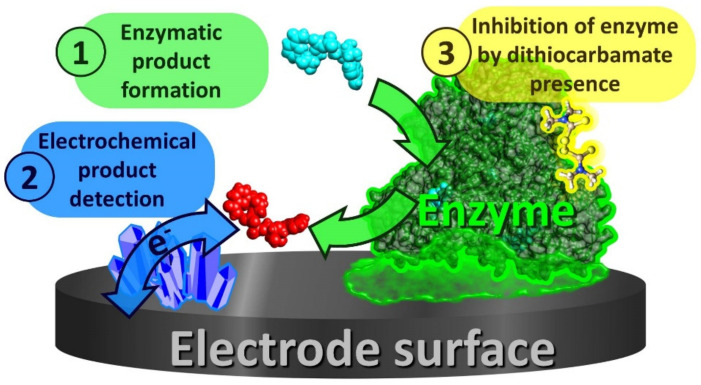
Generalized schematic of the operating principles of DTF-monitoring electrochemical biosensors. 1: During operation, enzymes immobilized at, or near an electrode, are supplied with their required cofactors and substrates which produce a constant rate of enzymatic product. 2: The electrochemical detection of the formed enzymatic products generates the biosensor’s signal. 3: The presence of DTF pesticides inhibits the enzyme activity, lowering the electrochemical signal to an extent proportional to DTF concentration.

In inhibition-based biosensors, the analytical signal is measured before and after exposure of the sensor to a sample containing the pesticide target; corresponding changes in signal are correlated to the concentration of the pesticide (Figure 5).

Depending on the precise enzyme and target under study, enzymatic inhibition is either reversible or irreversible and can follow either competitive, uncompetitive, noncompetitive, or mixed types of inhibition mechanisms. Understanding the kinetics of the enzymatic reaction and the inhibition mechanism is very useful for developing an efficient biosensor [76]. Inhibition-based biosensors rely mainly on optical and electrochemical detection methods, similar to those discussed in Section 2.3.1 and Section 2.3.2 above. A more detailed discussion can be found in [76] and [74], amongst others.

It is important to note that the enzymes employed are not inhibited by a specific compound, but by a range of pesticides belonging to specific chemical groups, as well as by other compounds such as heavy metals for example. This lack of selectivity has prompted some skepticism regarding the analytical opportunities of inhibition-based enzymatic biosensors [84]. Nonetheless, the potential advantages of such analytical devices (particularly, portability, and fast sensor responses) seem to prevail, making them suitable as rapid screening and alert systems. Consequently, these biosensors continue to attract interest. To address the lack of selectivity, multiplexed sensors coupled with chemometric analysis, genetically modified enzymes, as well as novel enzymes, e.g., extracted from extremophilic microorganisms, with different substrate specificity and inhibitor profile are continuously explored.

#### 2.4.1. Examples of Biosensors for the Determination of DTFs

For the detection of DTFs, several electrochemical biosensors have been developed as presented in Table 3 and detailed further below.

In a departure from most biosensor configurations, a whole-cell potentiometric sensor was developed using mammalian N2a (murine neuroblastoma) and Vero (green monkey kidney epithelial) cells entrapped in alginate beads, attached to a silver working electrode [73]. Detection of propineb in this format using the N2a cells was attributed to inhibition of membrane-bound acetylcholinesterases expressed by the mammalian cells and subsequent influence on the membrane polarization of these cells; a similar response was separately attributed to binding of the zinc-centered zineb to a zinc ion channel protein expressed by the Vero cells.

Laccases (polyphenol oxidase, EC 1.10.3.2) catalyze the oxidation of a wide range of aromatic organic compounds, including diphenols in the presence of molecular oxygen. The activity of laccase from *Trametes versicolor* is inhibited by ziram, but also by carbamate insecticides such as methomyl, pirimicarb, formetanate, carbaryl, and carbofuran [77,85,86] and by arsenate and arsenite. [87], among others. In a biosensor for the detection of ziram, laccase was dropcasted on a graphene doped carbon paste electrode, coated with a film of Prussian Blue [77]. When the biosensor was inserted in a solution of 4-aminophenol, upon sweeping the potential from +0.3 V to −0.1 V, 4-aminophenol was electrochemically reduced to an imine-quinone intermediate, that was further transformed into p-quinone in a reaction catalyzed by laccase. The cathodic peak current at −0.05 V due to the reduction of p-quinone, formed in the enzymatic reaction was proportional with laccase activity. Based on this principle, the biosensor enabled the analysis of ziram with a detection limit of 0.002 ppm, in a linear range between 2.49 × 10^−8^ M and 5.66 × 10^−7^ M. Several carbamate pesticides were also determined with detection limits of 0.001 ppm (carbaryl), 0.007 ppm (pirimicarb), 0.013 ppm (formetanate), and 0.022 ppm (carbofuran).

Tomato and potato extracts, spiked with pesticides at two concentration levels, were analyzed with the biosensor. Remarkably, a Quick, Easy, Cheap, Effective, Rugged, and Safe—QuEChERS method was used for pesticides extraction from the vegetable samples. The recoveries for ziram were 97.6–101.1% while for the other pesticides were 90.2–100.3%, indicating a satisfactory accuracy of the biosensor. In addition, the biosensor had a good reproducibility (RSD = 5.0%, *n* = 4 sensors) and repeatability (RSD = 1.8%, 2.4% for intra-day and inter-day repeatability) and was stable for 20 days. The selectivity study has focused on vitamins contained by tomatoes and potatoes (beta-carotene, i.e., pro-vitamin A, thiamine, i.e., vitamin B1 and ascorbic acid, i.e., vitamin C). Significant interferences, i.e., amounting to 11.4–13.2% of the analytical signal were found for beta-carotene and ascorbic acid, when tested at very high concentrations that were 10 times higher than those of spiked pesticides.

This report on a laccase-based biosensor provides a good illustration of both the potentialities and the challenges for the electrochemical biosensors for pesticides that rely on enzyme inhibition. The good sensitivity of the biosensor was attributed in part to the characteristics of the electrochemical transducer, obtained from carbon paste containing 20% graphene flakes and modified with an electrodeposited film of Prussian Blue. The graphene flakes had a thickness of few layers, a length of 500 nm–1.5 µm and contained 12.4% oxygen, 87.0% carbon, and 0.5% nitrogen. Modification of the carbon paste with this type of graphene improved the speed of the charge transfer in the electrochemical reduction of 4-aminophenol to the imine-quinone intermediate. Further modification of the electrode with Prussian Blue enhanced the cathodic current by approximately 27%. The use of nanomaterials to improve the conductivity and enhance the active surface area of the base transducer in biosensors is common nowadays, with more nanomaterials explored each day. Nonetheless, the characteristics of these nanomaterials vary in large limits, depending on the method used for obtaining them which dictates properties such as the degree of oxidation, number of stacked layers, size and possible contaminants, all influencing the electrochemical characteristics of the modified electrodes. While in this 2013 report [77] laccase was simply adsorbed on the electrode, controlled immobilization of laccase was also demonstrated in inhibition based biosensors for arsenate and arsenite [87]. Anthracene moieties covalently bound to multi-walled carbon nanotubes anchor laccase in a controlled manner, with the copper center of laccase oriented towards the electrode surface, thus allowing for direct electron transfer from enzyme to the electrode for the catalytic oxygen reduction. This immobilization approach also minimizes potential interferences due to chloride [87]. It is therefore reasonable to expect that better, more sensitive and selective inhibition-based biosensors can be obtained by exploiting the knowledge accumulated so far with regards of different nanomaterials and their coupling with enzymes and other modifiers. With regards to practical applications, the report of Oliveira et al., [77] emphasized the necessity of application-targeted investigations of possible interferences. Furthermore, QuEChERS methods have all the advantages for sample extraction denoted by their name but require lab-dedicated equipment. Sample pre-treatment remains therefore a bottleneck for the development of applications for in-field screening of DTFs and other pesticides. Moreover, definitive proof of biosensor accuracy should be obtained by comparing the results obtained with the biosensor with a standard confirmatory method or by using certified materials.

**Figure 6:**
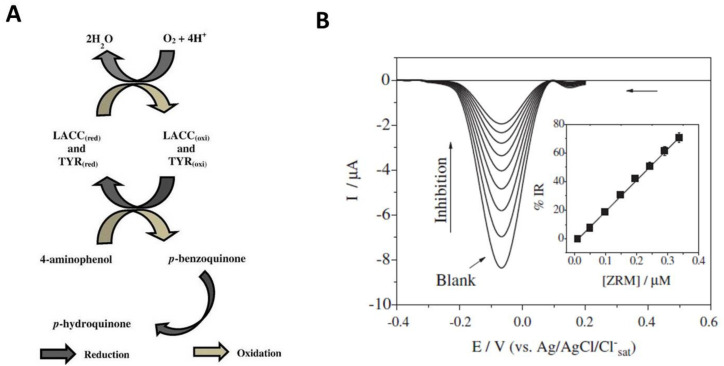
(**A**) Principle of DTF detection with a bi-enzymatic biosensor based on laccase and tyrosinase. (**B**) Magnitude of cathodic currents recorded with the bi-enzymatic biosensor for the reduction of p-benzoquinone to p-hydroquinone by square wave voltammetry, reported in [79]. Reproduced from [79] with permission.

In addition to laccase, tyrosinase is another enzyme that is inhibited by DTFs. Tyrosinase (EC 1.14.18.1, monophenol, o-diphenol: oxygen oxidoreductase) catalyzes the oxidation of monophenols to o-diphenols, as well as the further oxidation of diphenols to their corresponding quinones. Tyrosinase is also inhibited by atrazine, hydrazines, and cyanide. In electrochemical biosensor investigations, tyrosinase was coupled with laccase in a biosensor for the detection of carbamate pesticides, achieving higher sensitivity than when each enzyme was used alone [79]. The mono and bi-enzymatic laccase biosensors were applied for the determination of ziram, as well as the carbamate pesticides carbofuran, formetanate carbaryl, and propoxur in spiked vegetables (tomato, potato) and citrus fruit (lemon, tangerine, and orange). According to the principle depicted in Figure 6A, 4 amino-phenol was used as an enzymatic substrate that was converted into p-benzoquinone under the catalytic action of tyrosinase and laccase. The magnitude of the cathodic current due to the reduction of p-benzoquinone to p-hydroquinone was correlated with enzyme activity. The cathodic current decreased in the presence of ziram in a concentration dependent manner as depicted in Figure 6B.

The recovery results from fungicide-spiked vegetables and citrus fruit determined with the biosensors reported in [77,79] were in the range 90.2–101.1%, emphasizing the accuracy of the biosensors and supporting the feasibility of inhibition-based biosensors in detecting ziram and other pesticides in real samples.

Aldehyde dehydrogenase (E.C. 1.2.15, AlDH) catalyzes the transformation of aldehydes to the corresponding carboxylic acids in the presence of the enzymatic cofactor nicotinamide adenine dinucleotide (NAD^+^) or nicotinamide adenine dinucleotide phosphate (NADP^+^):

$$\mathrm{Aldehyde}+{\mathrm{NAD}}^{+}+H_{2}O\;\begin{array}{c} \mathrm{ALDH}\\ ⇋ \end{array}\;\mathrm{Carboxylic}\;\mathrm{Acid}+\mathrm{NADH}+H^{+}$$

In the human body, aldehyde dehydrogenase, more specifically ALDH2 is behind the connection between the exposure to pesticides and Parkinson’s disease. Aldehyde dehydrogenases, in general, are inhibited by a whole range of compounds, including dithiocarbamate fungicides, benzimidazole fungicides and some heavy metals [88].

In biosensors, measuring the activity of aldehyde dehydrogenase is achieved by determining the reduced cofactor, NADH, formed in the enzymatic reaction. DTFs inhibit the activity of ALDH, causing a decrease in the amount of NADH. The reduced cofactor can be sensitively detected by electrochemical oxidation at the surface of carbon electrodes. Furthermore, the electrochemical detection of NADH is efficiently accomplished using electrochemical mediators or various carbon nanomaterials such as carbon nanotubes, graphene, nanofibers, etc., which decrease the potential required for NADH oxidation [89]. By measuring at low potentials, close to 0 V, the risk of potential interferences from other electrochemically active compounds in samples is minimal, supporting the accuracy of the detection.

Noguer et al. and others have described several biosensors for the detection of dithiocarbamate fungicides, based on the inhibition of aldehyde dehydrogenase from baker’s yeast [78,80,81,82,90]. The equilibrium of the enzymatic reaction favors the reactants’ side, consequently, high concentrations of cofactor, alkaline pH, or coupled enzymatic reactions are used to shift the equilibrium of the enzymatic reaction towards the products side and achieve quantitative conversion of aldehydes and the NAD^+^ cofactor. A successful approach for the highly sensitive detection of fungicides, such as maneb and zineb [81,82], was to use a second enzyme, either NADH oxidase or diaphorase in order to convert very fast NADH, back to the oxidized form NAD^+^.

For example, the determination of maneb and zineb was achieved with a bi-enzyme biosensor comprising aldehyde dehydrogenase and diaphorase [90], according to the following scheme:

(1) $$\mathrm{Propionaldehyde}\;+\;{\mathrm{NAD}}^{+}\;+{\;H}_{2}O\begin{array}{c} \mathrm{ALDH}\\ ⇋ \end{array}\;\mathrm{Acetic}\;\mathrm{acid}+\mathrm{NADH}+H^{+}$$

(2) $$\mathrm{NADH}+{ [\mathrm{Fe}{\left(\mathrm{CN}\right)}_{6}]}^{3-}\;\overset{\mathrm{Diaphorase}}{\to }{\mathrm{NAD}}^{+}+{ [\mathrm{Fe}{\left(\mathrm{CN}\right)}_{6}]}^{4-}+H^{+}$$

(3) $${ [\mathrm{Fe}{\left(\mathrm{CN}\right)}_{6}]}^{4-}\;\overset{E\;>\;E0\prime }{\to }{ [\mathrm{Fe}{\left(\mathrm{CN}\right)}_{6}]}^{3-}+e^{-}$$

The NADH produced in the enzymatic reaction catalyzed by aldehyde dehydrogenase (1) is oxidized back to NAD^+^ by diaphorase, in a reaction where ferricyanide acts as electron acceptor (2). (3), the ferrocyanide formed in reaction (2) is oxidized at the surface of a Pt electrode, polarized at +0.1 V. The magnitude of the anodic current from (3) is proportional to the activity of aldehyde dehydrogenase in the reaction medium.

**Figure 7:**
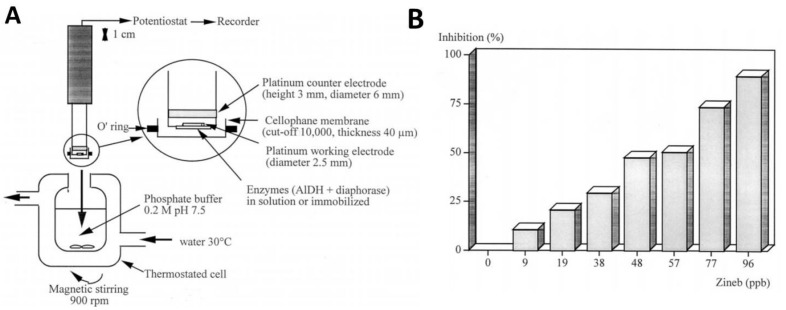
(**A**). Experimental setup used for the bi-enzymatic sensor for maneb and zineb. (**B**). Response of the biosensor at different concentration of zineb, expressed as percentage of the signal in the absence of the inhibitor. Reproduced from [90], by permission.

The enzymes were used either free in solution or immobilized in a matrix of PVA-SbQ, being retained at the surface of a Pt electrode with a cellophane membrane (Figure 7A). Enzyme immobilization in PVA-SbQ proved advantageous: after incubation with the pesticide solution for 10 min, the biosensor enabled detection as low as 1.48 ppb (maneb) and 9 ppb (zineb) [90] (Figure 7B). It should be highlighted here the approach used to solubilize zineb in alkaline medium in the presence of EDTA, thus transforming it into a disodium salt, known as nabam. Nabam is another DTF that inhibits aldehyde dehydrogenase.

The use of more stable NADH oxidase instead of diaphorase and disposable screen-printed electrodes instead of Pt electrodes were reported to simplify and reduce the costs of the enzymatic biosensor [82].

While achieving limits of detection in the ppb range, these devices were demonstrated exclusively with standard solutions of pesticides in buffer, with no testing of real samples.

The biosensors based on the inhibition of aldehyde dehydrogenase were developed more than 13 years ago. Re-starting the research efforts in this direction can bring improved performances and wider applications, considering the growing use of nanomaterials in enzymatic inhibition-based biosensors in the last years [75,91] and the opportunities brought by novel enzymes, isolated from various sources. These enzymes have potentially different substrate specificities and inhibitor profiles (discussed below).

**Figure 8:**
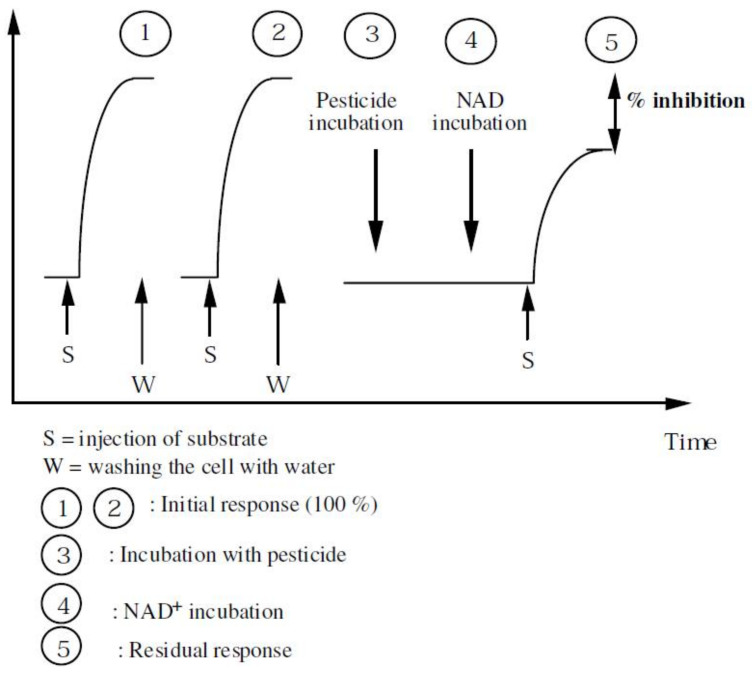
Typical experimental protocol for an amperometric biosensor for DTF based on aldehyde dehydrogenase. Reproduced from [78], by permission.

The critical factors affecting the analytical performance of the inhibition based enzymatic biosensors are: the amount of enzyme, the incubation time, the design of the device and of the sensing layer, including the matrix and type of immobilization of the enzyme. In general, as summarized in Table 3, the enzymes were entrapped in polymers by photopolymerization (e.g., in PVA-SbQ), adsorbed on electrochemically deposited films of Prussian Blue or were cross-linked with glutaraldehyde in a matrix of bovine serum albumin. The incubation time with the fungicide was less than 20 min. The design of the test must consider the reversibility and the mechanism of inhibition. For example, aldehyde dehydrogenase inhibition by dithiocarbamate fungicides is competitive versus the cofactor NAD^+^ and is irreversible. Therefore, the analytical signal is registered first in the absence of the fungicide, to get a reference signal, then the NAD^+^ is eliminated by washing and the signal is recorded in the absence of the cofactor. Next, the biosensor is incubated with the fungicide, followed by a short incubation with NAD^+^ and by the addition of the substrate, before recording the final signal. The biosensor is discarded after this, since the original activity of the enzyme cannot be restored (Figure 8).

These steps require an adequate operational stability of the sensor and appear to be too complex to be compatible with in-field testing. A possible solution to circumvent such complexity can be the use of magnetic beads with immobilized enzymes. A magnetic field is applied to “immobilize” the enzyme at electrode surface [92], the enzyme-modified magnetic particles are discarded after the test and the electrode is used for the next measurements.

From a practical application point of view, commercializing such biosensors would require adequate storage and operational stability and production by methods compatible with mass-production, simple use and relatively short analysis time.

With regards to the storage stability, enzymes from extremophilic sources, able to operate in a wider range of temperatures than their mesophilic counterparts, are a viable choice as specific receptors in the biosensor.

The following section details some examples of extremozymes, most promising with regards to stability and therefore with real potential to meet the requirements to be applied in biosensors for practical applications.

#### 2.4.2. Extremozymes as Potential Biorecognition Elements in Biosensors for DTFs

Extremophilic microorganisms constitute a recently exploited reservoir of enzymes stable under various conditions. These microbes developed a particular proteome presenting specific structural and functional features in order to cope with extreme temperatures, hydrostatic pressure, alkaline and acid pHs, high salinity, and radiations [93]. Therefore, for the last few decades, a series of enzymes (extremozymes) were investigated and used for improved industrial processes. Among extremophiles, thermophilic and hyperthermophilic bacteria and archaea represent the most abundant source of extremely stable enzymes that are also resistant to various chemical agents and extreme pH values [94].

Aldehyde dehydrogenases (ALDH, E.C 1.2.1.3) are one of the important yet little explored biocatalysts in biotechnologies and biosensing [95,96]. The inhibition pattern of these extremozymes was poorly studied, in particular the effect of dithiocarbamate derivates known as ALDH inhibitors on their NAD(P)-dependent activity [88]. Nonetheless, the high stability of these enzymes from thermophilic [97], psychrophilic [98], and halophilic microorganisms [99], over a broad pH and temperature range both in solution and immobilized, recommends them as putative enhanced biocatalysts for commercial operations.

Among this class of enzymes from hyperthermophiles, the bifunctional aldehyde dehydrogenase from the archaeon *Pyrococcus furiosus* appeared to be extremely stable, up to 100 °C, and highly active between pH 9.4 and 10.2 at 80 °C [100], while the heterologous ALDH from the thermoacidophilic archaeon *Sulfolobus solfataricus P2* also stable at high temperatures optimally catalyzed the NAD^+^-dependent aldehydes oxidation at 70 °C and pH 6.5 [101]. ALDH from the thermophilic and alkaliphilic *Natronomonas pharaonis* isolated from a highly saline soda lakes in Egypt; thus, adapted to hypersaline conditions and high pH, showed an optimal temperature of 60 °C in the presence of 0.25 M NaCl at pH 8 [97]. This extremozyme also showed a high long-term stability, preserving its full activity after 24 h storage in various concentrations of NaCl and a specific activity of ~1 µmol·min^−1^·mg^−1^ when oxidizing acetaldehyde at 20 °C [97].

Scant information is available so far on cold-active aldehyde dehydrogenases, considering that psychrophilic and psychrotolerant microorganisms constitute an important source of stable enzymes that are highly active at low temperatures. Among important cold-active candidates, the ALDH from the psychrotrophic marine Antarctic *Flavobacterium frigidimaris KUC-1* (formerly *Cytophaga* sp.) presented a broad-range thermostability up to 60 °C, with optimal pH >10 [98]. This extremozyme preserved 70% of the activity when incubating at 45 °C for 2 h and has a half-like of 65 min at 50 °C, being showed a lower activation energy at 30 °C as compared to the mesophilic *Saccharomyces cerevisiae* ALDH [102], favoring catalysis at commonly used temperatures in applicative reactions [98].

Moreover, the homologous recombinant enzyme from the same family originating from the Antarctic *Flavobacterium PL002* strain was recently used in an electrochemical test and in a biosensor for the detection of benzaldehyde [89,103]. The enzyme has wide substrate specificity and was shown to be inhibited by thiram [104], thus it appears to be a good candidate as a biorecognition element in a biosensor for DTFs. Moreover, the cold-active PL002 ALDH had only 20% activity reduction after storage for 1 week at 4 °C (C. Purcarea, unpublished data), as an important advantage for biosensing.

Studies of *Rhodococcus* *sp. NI86/21* able to degrade the thiocarbamate herbicide S-ethyl dipropylcarbamothioate revealed the presence of a NAD^+^-dependent ALDH active on aliphatic aldehydes involved in this cytochrome P-450 related process [105]. Knowing that *Rhodococcus* genus contained widespread polyextremophilic actinobacteria able to survive within a 4 °C to 45 °C temperature range, high hydrostatic pressure, UV irradiation and osmotic stress [106,107], with an extended array of enzymes as putative candidates for environmental and biotechnological applications [108], further investigation of ALDHs from extremophilic *Rhodococcus* species could lead to developments in biosensing for pesticides detection.

Microbial laccases (E.C. 1.10.3.2) constitute currently used sensing biocomponents for pesticides detection [85,109]. During the last decades, the characteristics of a large variety of native and recombinant laccases from thermophilic, psychrophilic, and alkaliphilic bacteria and fungi were reported [110]. The enzyme from the alkalotolerant gamma-proteobacterium JB isolated from industrial waste water, optimally active at 55 °C and pH 6.5, was 100% inhibited by 3.5 mM diethyldithiocarbamate when using syringaldazine as substrate, with a Ki of 0.163 mM, an effect reversed by 1.5 mM CuCl_2_ [111]. Meanwhile, in the case of the thermostable laccase from *Streptomyces lavendulae REN-7*, only a slight inhibition (14%) by sodium N,N-diethyldithiocarbamate trihydrate was observed [112].

Although the inhibitory effect of dithiocarbamate derivates was not investigated for a large variety of these extremozymes, functional studies of laccases from extremophilic microorganisms revealed their high thermal stability and a wide pH interval for catalysis that makes them good candidates for enhanced biosensing components for pesticides monitoring.

#### 2.4.3. Challenges in the Application of Biosensors for DTFs Determination in Real Samples

Most challenges to be solved in order to apply the biosensors for the determination of DTFs in real samples are common with those observed for other pesticides [113], namely, (i) improving the sensitivity, storage stability, reproducibility, and robustness of the sensing device, (ii) demonstration of selectivity in complex matrices, (iii) simplification of sample pre-treatment, and (iv) development of adequate working protocols to allow real time, on field analysis.

An additional hurdle though compared to other types of pesticides that were preferentially studied, is the lack of specific bioreceptors such as antibodies or aptamers and the lack of sample extraction cartridges based on molecularly imprinted polymers [114].

With regards to the sensitivity of enzymatic biosensors, bi-enzymatic devices, such as those based on laccase/tyrosinase and those combining aldehyde dehydrogenase with NADH oxidase or diaphorase, lead to enhanced performance for DTFs detection compared to mono-enzymatic ones. However, complexity, costs, and finding operational conditions that represent a good compromise for both enzymes have to be weighed against the increase in sensitivity. Rational biosensor design, including the controlled immobilization of enzymes and the use of well-characterized nanomaterials and modifiers provides a wealth of possibilities for biosensors for pesticides with improved characteristics [75,91,115].

As with all enzyme-inhibition based biosensors, the selectivity has to be accurately evaluated in accordance to the targeted application. For example, the sensitivity of laccase, tyrosinase and aldehyde dehydrogenase enzymes to several DTFs and other inhibitors can be exploited to develop screening-type systems, alerting on possibly contaminated samples that should be analyzed further by standard methods. Alternatively, sensors arrays such as bioelectronic tongues including several enzymes with different susceptibilities to DTFs, coupled with chemometrics for data analysis can be envisaged for the selective detection of specific compounds, similar to other devices described in literature [116].

Use of enzymes with improved stability, genetically modified enzymes or newly discovered enzymes with different inhibition profiles, inclusion of nanomaterials and stabilizers and fabrication by methods compatible with mass-production are expected to solve issues related to sensitivity, stability, reproducibility and robustness.

The low solubility of DTFs in water is a further complication for sample preparation as are the extraction steps needed to ensure quantitative recovery from food matrices. The development of portable biosensor-based devices can be envisaged however, for the fast screening of DTFs on the surface of intact fruits and vegetables and wherever DTFs can be converted easily to soluble salts. In this sense, a wearable glove biosensor, based on the inhibition of acetylcholinesterase that was recently demonstrated for the detection of organophosphorus pesticides on the surface of intact vegetables [117] can serve as a model.

Comparison with standard confirmatory methods, lacking in biosensor reports will go a long way to support the feasibility of such devices and is anticipated to encourage research in this direction.

## 3. Conclusions and Perspectives

Despite the large use of DTFs in agriculture, research in the field of analytical methods dedicated to these fungicides has been very limited compared to other classes of pesticides. Today, like 20 years ago [72], most standard methods for the analysis of DTFs remain based on the degradation of fungicides to CS_2_ and measuring the resulting amount of CS_2_, which does not make it possible to discriminate between various DTFs with different toxicities.

A small number of chromatographic methods coupled with mass spectrometric detection enable the separation and detection of DTFs from different groups, i.e., propylene-bis-, ethylene-bis and dimethyl-dithiocarbamates. Moreover the specific detection of metal containing DTFs was achieved by coupling chromatographic separation with atomic absorption spectroscopy. Recent advances in chromatography based approaches mainly concerned sample extraction procedures.

Most research efforts in the last years were concentrated on the SERS-based detection of DTFs, targeting: (i) portable devices, (ii); simple and effective sampling procedures; and above all (iii) the discovery of new cost-effective Ag, Au, and Cu-based nanomaterials and composites as SERS substrates for the highly sensitive detection of DTFs and thiram in particular. Various nanomaterial morphologies, modifiers for specific anchoring the analyte to the hot spots, 2D and hierarchical 3D nanostructures have been investigated aiming for high sensing area with a high density of “hot spots” for enhanced signals. In line with the current trends, significant advances were noted for the “paste and peel” sensors based on flexible supports, which could be brought in close contact with the sample surface and thus play a double role, i.e., sampling and sensing, to enable the fast, in situ analysis of thiram. The detection of thiram, as a prominent example of DTFs was demonstrated in a variety of spiked samples, including soil, strawberries, tomato, cucumber, water, etc., for which satisfactory recoveries were calculated. Without any doubt, considering the effervescence of research on this topic, the progress with portable devices and the efforts towards enhancing the accuracy of the detection (e.g., by including internal standards, using chemometrics for analyzing the data etc.), SERS-based methods have a very high potential to achieve in situ selective detection of specific DTFs.

In addition to SERS, many assays based on optical and electrochemical detection have been developed. Oftentimes the accent was placed on developing simple and low cost procedures. However, the selectivity of most devices was not unambiguously demonstrated, and their accuracy remains to be confirmed by comparison with standard methods.

While biosensors appear as an attractive alternative to separation-based methods with their promise for fast, selective, cost effective, portable, and simple detection, the detection of DTFs was rarely explored. This is in part because antibodies, aptamers, and molecularly imprinted polymers for DTFs are not available commercially as for other pesticides. The few reports on biosensors for DTFs based on the inhibition of laccase, tyrosinase or aldehyde dehydrogenase (some more than 10 years old) emphasize their capability to reach detection limits compatible with practical applications. The development of biosensors for DTFs can be fast tracked by exploiting the knowledge and adapting concepts from biosensors for other pesticides, e.g., the widely studied organophosphates and carbamates that inhibit cholinesterases.

More specifically, controlled immobilization of enzymes, use of nanomaterials to enhance the electrochemical signal, embracing the trend for flexible devices used for both sampling and detection can potentially lead to highly sensitive biosensors for DTFs for in situ detection applications. For example, many opportunities are anticipated for devices such as biosensor swipes or gloves for thiram detection on the surface of fruits and vegetables.

Considering that laccase, tyrosinase and aldehyde dehydrogenase are inhibited in different proportions not by a single fungicide, but by a group of DTFs as well as by several other compounds, the most direct application of enzyme-based biosensors is as alert systems. Selective detection of a particular compound might be attempted in the future with bioelectronic tongues. Enzymes obtained by engineering approaches, with improved selectivity and stability, or new enzymes isolated from extremophiles, with new substrate specificity profile and enhanced stability can contribute to meet the requirements for practical applications of biosensors in DTFs analysis.

Obviously, there is a high need of stable, specific receptors such as aptamers or molecularly imprinted polymers that could simplify the analysis of DTFs and assist not only with detection but also with sample extraction and cleaning. Two main strategies are currently pursued with sample pre-treatment: development of QuEChERS methods, suitable for laboratory-based analysis of any type of sample and fast methods, such as “paste and peel”,“swipe”, etc., mainly intended for fast, in situ sampling, resulting in flexible films adhering to sample surfaces that collect the contaminants and are afterwards directly used for sensing by SERS. While acknowledging limitations due to the low solubility in water and organic solvents of most DTFs, further progress enabling fast, quantitative recovery of DTFs is expected for specific applications, such as the detection of thiram from the surface of intact fruits and vegetables.

In summary, although various approaches are available for the detection of DTFs, selectivity remains a critical issue to be addressed in a more detailed and application-oriented manner in the coming years. Clearly, there are many analytical opportunities ahead in the analysis of DTFs and the field is one deserving far more concentrated research efforts.

## Figures and Tables

**Table 1:** Direct electrochemical detection: selectivity via catalysis and/or electrode potential control.

Dithiocarbamate Pesticides Investigated	Electrode Surface (Catalyst/Modifier) ^a^	Real Samples Investigated	Signal Basis ^b^	L.O.D. (Analytical Ranges Reported)	Ref.
Ziram	Polished silver solid amalgam electrode	Spiked river waters	SWV	0.24 µM	[38]
Thiram	Hg		CS-DPV peak at −0.55 V vs. Ag/AgCl	0.12 μM	[39]
Thiram	Rotating gold disk electrode	Commercial formulations; spiked water samples	Ads-LSV, peak at +1.4 to +1.5 V vs. Ag/AgCl	16 nM	[40]
Thiram Disulfiram	Graphite-PTFE composite electrode	Extracts of spiked strawberry samples	Ads-LSV, peaks at +0.85 V vs. SCE	Thiram: 54 nM (0.2 to 1 µM) Disulfiram: 20 nM (0.2 to 1 µM)	[41]
Thiram Disulfiram	Graphite-PTFE composite electrode	Spiked tap and well water samples	FIA-CA at +1V vs. Ag/AgCl	Thiram: 43 nM (0.1 to 1 µM) Disulfiram: 20 nM (0.1 to 1 µM)	[42]
Ziram	Hg	Extracts of spiked rice samples	CS-DPV	32 nM i.e., 10 ppb	[43]
Zineb	Hg		AdSV, cathodic peak at −0.455 V vs. Ag/AgCl	1 nM	[44]
Carbathion, Ferbam, Nabam, Thiram, Thiuram, Zineb, Ziram	Carbon paste electrode -Fe(II)metallophthalocyanine composite		Ads-LSV	Ranged from 10 nM (carbathion) to 200 nM (Thiuram)	[45]
Nabam	GCE, modified with Co(II) phthalocyanine and carbon ink		LSV, peak at −0.2V vs. Ag/AgCL	28.8 nM	[46]
Thiram	GCE	Commercial formulations; plant sample extracts exposed to thiram	SWV at +0.34 V vs. Ag/AgCl	n.r.	[47]
Carbathion	GCE		CV, peak forming at +1.46 vs. Ag/AgCl	9.3 μM (132 μM to 224 μM)	[48]
			SWV, peak forming at +1.46 vs. Ag/AgCl	85 nM (2 μM to 7.7 μM))	
			FIA-CA potential of +1.3 V vs. Ag/AgCl	10 nM (1.2 μM to 6 μM)	
Ziram	Hg	Extracts of spiked vegetable samples	SWV, −1.1V vs. Ag/AgCl.	23 nM (33 to 328 nM)	[37]
Thiram	Copper-mercury amalgam electrode	Spiked river water samples	CS-SPV, peak between −0.59 and −0.8 V vs. Ag/AgCl	16 nM	[49]
Propineb	Carbon-paste electrode (Cu^2+^-enriched montmorillonite)	Commercial formulation	Ads-SWV, peak at ~−0.1V vs. SCE	1 μM	[50]
Mancozeb	BDD		PAD at +0.3V vs. Ag/AgCl)	0.514 µM (40 to 650 µM)	[36]
Mancozeb	GCE	Commercial formulation	Ads-SWV, peaks forming at −0.7V vs. Ag/AgCl	7 µM	[51]
Ziram	BDD	Spiked river water samples	FIA-CA at +0.55 V	2.7 nM	[52]
Maneb	BDD	River water	DPV peak at +0.9V vs. Ag/AgCl	24 nM (80 nM to 3 µM)	[53]
Mancozeb	Single-crystal (Au(111) and Au(110)		Ads-LSV, peaks at −0.6 to −0.96V vs. Ag/AgCl	Au(110): 100 nM Au(111): 500 nM	[54]
Mancozeb	Gold electrode modified with Poly (3,4-ethylene dioxythiophene), multi-walled carbon nanotubes, and gold nanoparticles	Water	CV, anodic peak +0.65 V vs. Ag/AgCl	5 μM	[55]
Thiram	Carbon paste electrode modified with zeolite	Aqueous extracts of fruit juices	DPV, anodic wave at +0.70V vs. Ag/AgCl;	4 nM (14 nM to 4.2 μM)	[56]
Thiram	Platinum, modified with silver nanoparticles	Tap, canal, and river water	DPV and CV	0.731 μM or 0.18 ppm	[57]
Thiram	GCE (dissolved Zn^2+^ and Cu^2+^ cations)	River water	CS-LSV: −1.330 V vs. Ag/AgCl for Zn-Thiram; +0.020V for Cu-Thiram complexes.	n.r. (5 to 50 μM)	[58]

^1^ Where possible, analytical parameters have been standardized: limits of detection and analytical ranges of reported sensitivities are standardized to mol/L (M) units and detection sensitivities of amperometric and voltametric signals (i.e., peak currents and or response currents) were standardized to µA/µM values; ^a^—Electrode surfaces:BDD: Boron-doped diamond; GCE: Glassy carbon electrodes; Hg: Mercury (Drop) Electrodes; PTFE: poly(tetrafluoroethylene); ^b^—Signal basis:Ads-: Adsorptive (prefix); AS—Anodic Stripping (prefix); CS—Cathodic Stripping (prefix); FIA—Flow-injection analysis (prefix);CA: Chronoamperometry CV: Cyclic Voltammetry; DPV—differential pulse voltammetry; LSV: Linear Sweep Voltammetry; PAD: Pulsed Amperometric Detection; SCE: Saturated calomel electrode; SWV: Square-wave voltammetry; IUPAC designations of commercial dithiocarbamates studied in published articles: Carbathion: sodium N-methyldithiocarbamate (also known as metam sodium); Disulfiram: tetraethylthiuram disulfide; Diram: sodium N,N-dimethyldithiocarbamate; Ferbam: iron N,N’-dimethyldithiocarbamate; Maneb: Manganese ethylene-*bis*-dithiocarbamate; Mancozeb: 1,2-ethanedicarbamic acid, tetrathio- Manganese Zinc ethylene-*bis*-dithiocarbamate); Metam sodium: sodium N-methyldithiocarbamate; Nabam: sodium N,N’-ethylene-*bis*-dithiocarbamate; Propineb: Zinc propylene 1,2-*bis*-dithiocarbamate; Thiram: tetramethylthiuram disulfide; Thiuram: tetraethylthiuram disulfide; Zineb: zinc N,N’-ethylene-*bis*-dithiocarbamate; Ziram: N,N’-dimethyldithiocarbamate.

**Table 2:** Chromatography-coupled detection: selectivity via chromatographic separation of sample components.

DTF Investigated	Electrode Surface (Catalyst/Modifier)	Sample	Applied Potential	L.O.D.	Ref.
Thiram	CPE	Spiked river water	+1.1V vs. Ag/AgCl	2.07 µM,	[12]
Thiram Disulfiram	Composite PTFE-graphite paste electrodes	Spiked apple samples	+1V vs. Ag/AgCl	Thiram: 1.66 µM Disulfiram: 3.37 μM	[60]
Carbathion Thiram Zineb	GCE	Spiked fruit pulp samples	+1.1 V vs. Pd	0.7 μM Thiram: 1.5 μM Carbathion: 0.7 μM	[59]
Carbathion Mancozeb Propineb Ziram	not reported		+0.6V vs. Pd.	Carbathion: 31 nM Mancozeb: 7 nM Propineb: 26 nM Ziram: 26 nM	[10]
Thiram	GCE	Spiked tap water and beetroot juice	+1.4V vs. Ag/AgCl.	13.4 nM	[13]
Thiram, disulfiram	AuNP-SPCE	Spiked apple, grape and lettuce samples	+1.2 V vs. Ag/AgCl	Thiram: 91 nM Disulfiram: 0.56 µM	[14]

CPE: carbon paste electrode. GCE: glassy carbon electrode. AuNP-SPCE: Gold nanoparticle modified screen-printed carbon. PTFE: poly(tetrafluoroethylene).

**Table 3:** Examples of biosensors for DTF based on enzymatic inhibition.

Fungicide	Detection Method	Enzyme	Limit of Detection	Incubation Time	Reference
Ziram	Square wave voltammetry/GPE	LACC ^1^, adsorption on electrodeposited Prussian Blue film	0.002 ppm	15 min	[77]
Ziram	Square wave voltammetry/GPE	LACC-TYR-AuNPs -CS electrodeposited film	1 ppb	20 min	[79]
Maneb	Amperometry/Pt electrode	ALDH+DP, entrapment in PVA/SbQ	1.48 ppb	15 min	[81]
Zineb	Amperometry/Pt-sputtered SPCE	ALDH and NADH oxidase/entrapment in PVA/SbQ	8 ppm 8–80 ppb	5 min	[82]
MITC	Amperometry/MBRS SPCE	ALDH/entrapment in PVA/SbQ	100 ppm	10 min	[78]
Maneb and zineb	Chronoamperometry/MBRS-SPCE	ALDH/ entrapment in PVA/SbQ or cross-linking with glutaraldehyde	31.5 ppb–maneb 35 ppb-zineb	10 min	[80]
Propineb (and organophosphates)	Potentiometry/Ag coated with AgCl	Working electrode inserted into Calcium-alginate beads containing 5 × 10^4^ cultured N2a or Vero mammalian cells.	0.33 μM (Vero cells) to 1.65 μM (N2a)	2.5 min	[83]

^1^ Abbreviations: PVA-SbQ: poly(vinyl alcohol), bearing styrylpyridinium groups. MITC: Methyl Isothiocyanate. MBRS: Meldola Blue-Reinecke salt. GPE: Graphene doped carbon paste electrode. AuNO: gold nanoparticles. CS: chitosan SPCE: screen-printed carbon electrode. LACC: laccase. Tyr: tyrosinase. ALDH: aldehyde dehydrogenase. DP: diaphorase.

## Data Availability

No new data were created or analyzed in this study. Data sharing is not applicable to this article.
